# Effects of post-adulthood environmental hygiene improvement on gut microbiota and immune tolerance in mice

**DOI:** 10.1128/aem.02477-24

**Published:** 2025-03-06

**Authors:** Na Li, Mengjie Li, Honglin Zhang, Zhimao Bai, Zhongjie Fei, Yangyang Dong, Xinting Zhang, Pengfeng Xiao, Xiao Sun, Dongrui Zhou

**Affiliations:** 1 Key Laboratory of Child Development and Learning Science of Ministry of Education, Southeast University 12579 , Nanjing, China; 2 College of Food Science, Nanjing Xiaozhuang University 74587 , Nanjing, China; 3 Key Laboratory of Environmental Medicine Engineering of Ministry of Education, Southeast University 660287 , Nanjing, China; 4 State Key Laboratory of Bioelectronics, Southeast University 127382 , Nanjing, China; Norwegian University of Life Sciences, Ås, Norway

**Keywords:** gut microbiota, environmental cleanliness, chronic inflammatory dermatosis, cytokine

## Abstract

Changes in diet, cleanliness, stress, and exercise patterns may contribute to the disappearance of various gut microbes in humans who relocate to developed countries from developing countries. To explore the impact of environmental cleanliness on the gut microbiota, adult mice housed in a general animal room were divided into three groups. The control group was subjected to an unchanged living environment, SPF mice were moved to a specific pathogen-free (SPF) animal room with higher environmental cleanliness, and SPFL (specific pathogen-free specific with a fecal leakage grid) mice were moved to the SPF animal room and reared in cages with the function of preventing mice from eating feces as much as possible. Metagenome sequencing results showed that the gut microbial diversity decreased after the environmental change, accompanied by a substantial loss in gut microbiota, including genera known to have protective effects against allergies and those involved in short-chain fatty acid production. Additionally, the abundance of functional genes involved in short-chain fatty acid metabolism, amino acid synthesis, vitamin metabolism, flagellar assembly, and bacterial chemotaxis decreased. The environmental hygiene improvement also resulted in significant increases in total serum IgE, IL-4, IL-5, and IL-13 levels in mice with artificially induced chronic inflammatory dermatosis. Compared with SPF mice, preventing mice from eating feces as much as possible decreased the gut microbial diversity but did not markedly change functional gene expression or total serum cytokine levels.

**IMPORTANCE:**

Research has indicated that the human gut microbial diversity gradually decreases, while the prevalence of allergic diseases increases after movement from developing countries to developed countries. A healthy gut microbiota is necessary for proper human immune function. Movement from undeveloped to developed regions is often accompanied by an increase in environmental cleanliness. However, whether changes in environmental cleanliness are an important factor contributing to the decreased gut microbial diversity and increased prevalence of allergic diseases has not been reported. This study demonstrates the impact of increased environmental cleanliness on gut microbiota and susceptibility to allergic diseases and contributes to a better understanding of the increased incidence rate of various chronic diseases.

## INTRODUCTION

With the development of the economy and society, population mobility has become an increasingly common phenomenon (1). It has been reported that relocation from developing countries to the United States led to a decrease in gut microbial diversity (2, 3). Moreover, the longer the time since the relocation, the lower the gut microbial diversity (2, 3).

The development of chronic diseases, such as allergies, is closely associated with an imbalance in the intestinal microecology (4–7). Research has demonstrated that the risk of developing food sensitization in infants decreases by 55% with each quarterly increase in gut microbial richness (8). Several epidemiological studies have shown that relocation to developed countries is associated with an elevated incidence of allergic diseases, and the time of relocation to new areas positively correlates with the incidence rate (9). Therefore, it is important to explore the critical factors that contribute to the decrease in gut microbial diversity during relocation to developed countries for preventing and treating chronic diseases.

The process of human relocation often involves changes in multiple factors that lead to alterations in gut microbiota composition and diversity, such as diet, psychological stress, environmental cleanliness, and urbanization (2, 3, 10–15). Vangay et al*.* found that changes in diet can only partially explain changes in gut microbiota, while other factors such as exposure to stress, exercise, municipal drinking water, antibiotics, and changes in antiparasitic treatment may all affect the gut microbiota (2). It is difficult to identify critical influence factors through clinical or epidemiological investigation experiments.

Previous animal experiments have shown that exposure to low-cleanliness environments significantly increases gut microbial diversity (11, 16, 17). Following the administration of antibiotics, a low-cleanliness environment has been shown to facilitate the rapid restoration of gut microbial diversity in mice, irrespective of whether they are on a high-fat or low-fat diet (18). Does environmental hygiene improvement have a significant impact on the structural composition and function of intestinal microorganisms? There is currently no clear report on this scientific issue (2).

In the current study, we used C57 BL/6 mice as an animal model to explore the effects of environmental hygiene improvement on the gut microbiome. A low-cleanliness environment was simulated by adding soil to the bedding in a general animal room, while a high-cleanliness environment was simulated using a specific pathogen-free (SPF) animal room. In addition, cages designed to prevent mice from eating feces as much as possible were used to simulate strict fecal–oral separation in humans. All other variables like diet, age, genetic background, physiological status, and original gut microbiota were controlled. At 8 weeks of age, mice living in a low-cleanliness environment were transferred to a high-cleanliness environment. We sequenced the 16S rRNA gene and metagenome of fecal microorganisms to determine the effects of environmental hygiene improvement on gut microecology and functional genes in the mice. After artificially induced chronic inflammatory dermatosis, total serum IgE and cytokine concentrations were determined to analyze the immune response of the mice.

## MATERIALS AND METHODS

### Animals and experimental groups

Six-week-old C57 BL/6 breeding mice were purchased from B&K Universal Changzhou and raised in a general animal room, where soil was added to the bedding. The soil was collected from the top 10 cm of a farm ground (Table S1). Two females and one male were housed in a cage for breeding, and the female mice were placed in separate cages after pregnancy. The mice were bred and raised up to the third generation in this environment. Thirty 4-week-old third-generation mice were randomly divided into three groups (*n* = 10/group) and continued to be housed in this environment until 8 weeks of age, and fecal samples were collected. Two groups of mice were transferred to the SPF animal room on the day of sampling. Subsequently, the first group was housed in the general animal room with the environmental cleanliness conditions unchanged. The second group (SPF group) was moved to the SPF animal room and provided with SPF bedding. The third group was also transferred to the SPF facility but housed in specialized cages equipped with a fecal leakage grid, designed to minimize coprophagy, and referred to as the SPFL group. Fecal samples were also collected on days 7 and 21 post-movement (Table S2). All fecal samples were frozen at −80°C before DNA extraction.

### Skin sensitization and serum cytokine level analysis

At 21 weeks of age, which was the 13th week after movement, the three groups of mice were repeatedly treated topically with DNFB (2, 4-dinitrofluorobenzene) to induce chronic inflammatory dermatosis. DNFB is a hapten, and repeated skin stimulation with DNFB can create a model of chronic inflammatory dermatosis (19–22). We initially stimulated the skin of the ears and back skin of the mice with 2 µL of 0.15% DNFB dissolved in acetone/olive oil (3:1) on days 1 and 4 of treatment. The same areas were then treated with 0.2% DNFB on days 7, 10, and 13. Before treatment with DNFB on days 7, 10, and 13, the ear and back skin lesions were scored according to dryness, scab, and keratinization with the score ranging from 0 to 10 (23). Briefly, a score of 0 indicated an asymptomatic state, with the larger scores correlating with the development of more lesions. The mice were euthanized 24 hours after the final DNFB treatment, and whole blood samples were collected and processed for obtaining serum. Serum IgE levels were determined using the ELISA MAX Deluxe Set Mouse IgE (Biolegend, San Diego, CA, USA), and serum cytokine levels were determined using the LEGENDplex MU Th Panel kit (Biolegend) in strict accordance with the manufacturer’s instructions.

### 16S rRNA gene sequencing

Microbial DNA was extracted from fecal samples using the E.Z.N.A. Stool DNA Kit (Omega Bio-tek, Norcross, GA, USA) according to the manufacturer’s instructions. The V4 region of the 16S rRNA gene was amplified using primers 515F and 907R (24). Amplicons were extracted from 2% agarose gels after electrophoresis and purified using the AxyPrep DNA Gel Extraction Kit (Axygen Biosciences, Union City, CA, USA) according to the manufacturer’s instructions and quantified using QuantiFluor-ST (Promega, USA). The PCR product library was then paired-end sequenced (2 × 250) using the Illumina MiSeq platform. High-quality 16S rRNA gene sequences were obtained by removing low-quality sequences using Trimmomatic (version 0.32) (25) and chimeric sequences using UCHIME (version 4.2.40) (26). Processed sequences were used to define the OTUs using Usearch (version 7.1; http://drive5.com/uparse/) with a threshold of 97% similarity. Using the Ribosomal Database Project (RDP) Classifier (version 2.2; https://sourceforge.net/projects/rdp-classifier/), the taxonomic identities of the phylotypes were determined at a confidence threshold of 70%. The reads of each sample are presented in Table S2.

### Shotgun metagenomic sequencing

Metagenomic shotgun sequencing libraries were constructed and sequenced. Briefly, 1 µg of the genomic DNA from each fecal sample was sheared using the Covaris S220 Focused-ultrasonicator (Woburn, MA, USA), and fragments approximately 450 bp in length were used to construct sequencing libraries. The libraries were sequenced in a paired-end 150-bp (PE150) mode using the Illumina HiSeq X instrument. Trimmomatic (version 0.32; http://www.usadellab.org/cms/?page=trimmomatic) was used to remove adapter contaminants and low-quality reads (25). The Burrows–Wheeler Aligner (BWA, version: 0.6) MEM algorithm (parameters: -M -k 32 t 16; http://bio-bwa.sourceforge.net/bwa.shtml) was used to map the human genome (version: hg19); host genome reads were removed, and clean reads were obtained. Kraken2 (27) was used to annotate the taxonomy of the clean reads according to the customized Kraken database, and Bracken (version: 2.0.0; https://ccb.jhu.edu/software/bracken/) was used to estimate the abundance of the taxonomy.

MegaHit (version: 1.1.4) with “--min-contig-len 500” parameters (28) was used to generate a set of contigs of each sample from clean sequence reads, and Prodigal (version: 2.6.3) (29) was used to predict open reading frames (ORFs) of the assembled contigs. ORFs were clustered using CD-HIT (parameters: -n 9 c 0.95 G 0 M 0 -d 0 -aS 0.9 r 1) (30) and generated to a set of unique genes. The longest sequence of each cluster was the representative sequence of the unique-gene set. The Salmon software (31) was used to obtain the read number of each gene for calculating the gene abundance in the total sample. Blastx was used to search the unique-gene set against the KEGG database to identify proteins and retrieve their functional annotations.

### Data analysis and statistical tests

Data analysis using the 16S rRNA gene was performed as per reported methods (32). Briefly, each sample was rarefied to 46,117 sequences, and the QIIME software package (version 1.9.1) was used to analyze the α diversity and β diversity of the 16S rRNA gene. The β diversity obtained after shotgun metagenomic sequencing was analyzed using R (version 4.0.5). Specifically, the vegan package was employed for calculating beta diversity metrics. The vegdist() function was used to compute the Bray–Curtis dissimilarity index between samples, which was then visualized using principal coordinate analysis (PCoA) to explore the differences in the microbial community composition. The nonparametric statistical test of OTU/species/KO was performed using R (version 4.0.5), and the other tests were performed using SPSS (version 1.8.0). Random forest analysis of OTUs/species was performed using R (version 4.0.5) with the randomForest package. The analysis was performed with the default parameters, using 500 trees (ntree = 500) in the forest and the number of features randomly selected at each split set to the square root of the total number of features (mtry = sqrt(number of features)). During the analysis, rare features were not removed, and the data were not transformed or scaled. We used the mean decrease accuracy as the measure for feature importance. All settings were the default settings, and out-of-box (OOB) error was used to estimate the generalization error.

## RESULTS

### Environmental hygiene improvement decreases the gut microbial diversity and richness in mice

**Fig 1:**
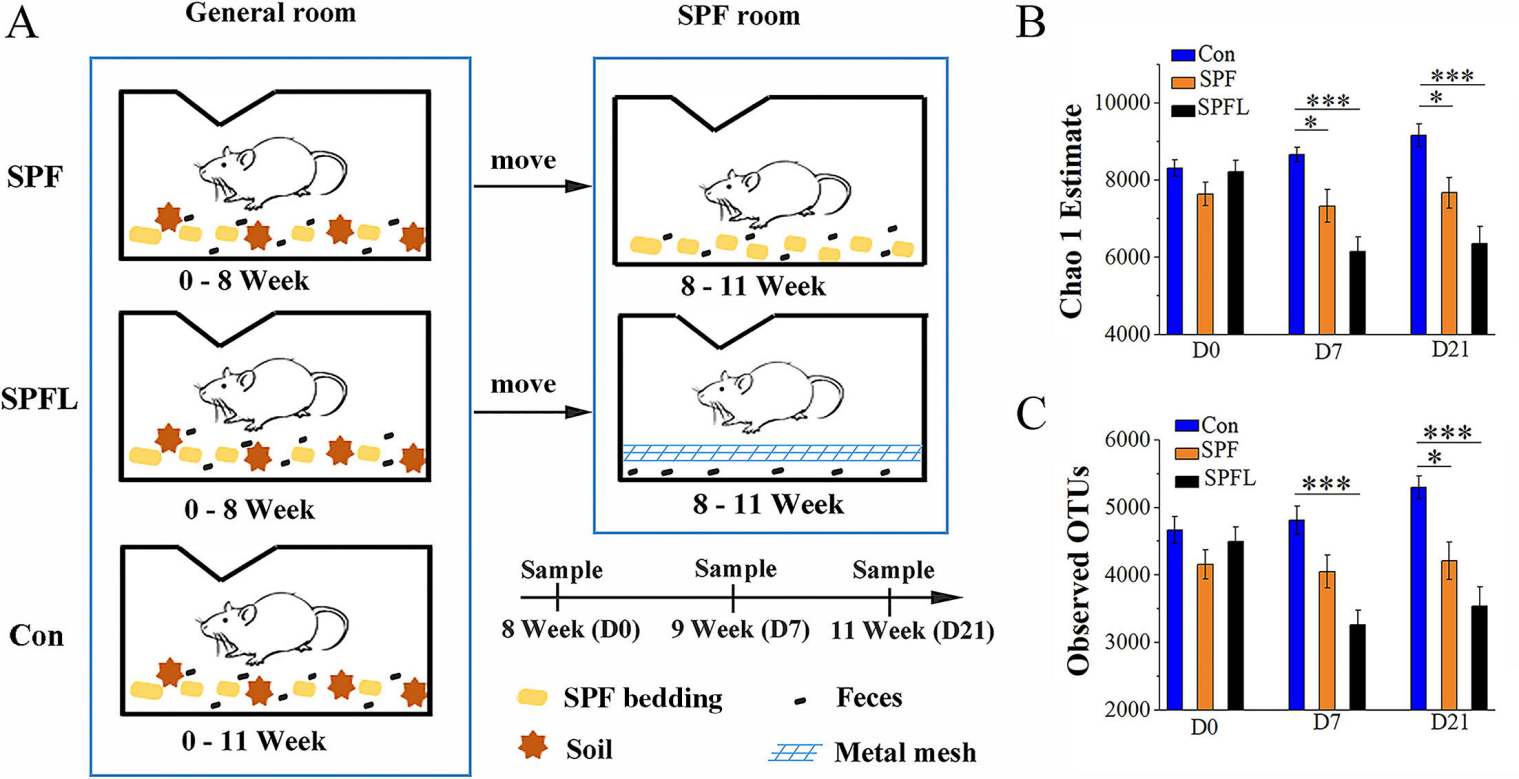
Effects of environmental hygiene improvement on the gut microbiota diversity and richness in mice. (A) Schematic mouse treatment and sampling timeline. A metal mesh was used to filter fecal leakage to prevent mice from touching and eating feces as much as possible. The control group (Con) was housed in the general animal room during the experiment, the SPF group was moved from the general animal room to the SPF animal room, and the SPFL (specific pathogen-free specific with a fecal leakage grid) group was moved to the SPF animal room and reared in cages with the function of preventing mice from eating feces as much as possible. D0 indicates the day of environmental hygiene improvement, D7 indicates the 7th day after environmental hygiene improvement, and D21 indicates the 21st day after environmental hygiene improvement. (B) Comparison of Chao1 estimates between different groups at the same time point. (C) Comparison of observed operational taxonomic unit (OTU) number between different groups at the same time point (*n* = 9 or 10; *P* values were based on ANOVA with Sidak’s *post-hoc* test (variance is homogeneous) or ANOVA with Tamhane *post-hoc* test (variance is uneven); *P < 0.05, **P < 0.01, and ***P < 0.001, Error bars represent the standard error of mean.

To determine the effects of environmental hygiene improvement on the richness and diversity of the gut microbiota in mice, we performed MiSeq sequencing to analyze the bacterial 16S rRNA gene V4 region of fecal samples before and after environmental hygiene improvement (Fig. 1A). We sequenced 89 samples and obtained 5,175,124 reads. Since the changes in gut microbiota with time are dynamic (33), in order to account for changes in the gut microbiota caused by environmental hygiene improvement, we compared the changes in the gut microbial diversity among different groups at the same time point (Fig. 1B and C). The results of the number of observed operational taxonomic units (OTUs) and Chao 1 abundance estimates showed that there was no significant difference in the gut microbiota diversity or abundance among the three groups on day 0, the day of environmental hygiene improvement (Fig. 1B and C; Table S3). However, on the 7th day, both the SPF and SPFL groups showed significantly lower Chao 1 abundance estimates than the control group, and the SPFL group showed significantly lower numbers of observed OTUs than the control group (Fig. 1B and C, Table S3). The results were similar on the 21st day compared to those on day 7 (Fig. 1B and C, Table S3). Similar results were obtained from the analysis of the PD index and the Shannon index (Fig. S1). By comparing the SPF group and the SPFL group, we observed a trend of decreased gut microbiota diversity on the 7th day following fecal leakage treatment (Fig. 1B and C, and S1; Table S3). Specifically, significant differences were found in the PD index and Shannon index (Fig. S13). On the 21st day after fecal leakage treatment, the gut microbiota exhibited a similar trend to that observed on day 7 (Fig. 1B and C, and S1; Table S3). Therefore, coprophagy in mice increases the diversity of the gut microbiota. Consequently, as environmental cleanliness increases, there is a gradient decline in gut microbiota diversity.

### Environmental hygiene improvement changes the gut microbiota composition in mice

**Fig 2:**
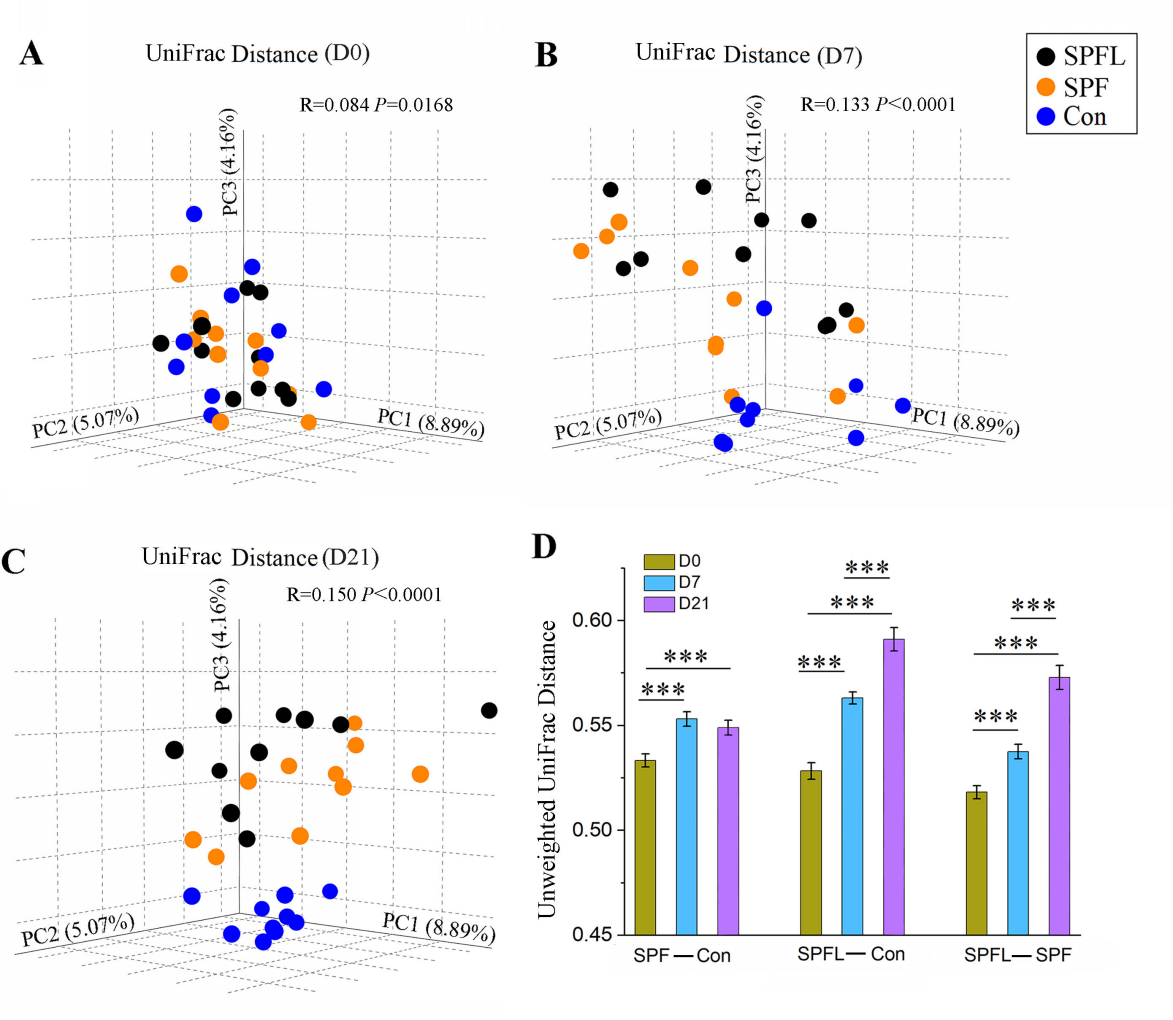
Effects of environmental hygiene improvement on the β diversity of gut microbiota in mice. (A, B, and C) Principal coordinate analysis (PCoA) of unweighted UniFrac distance of 16S rRNA gene sequences on the day of movement, day 7 after environmental hygiene improvement, and day 21 after environmental hygiene improvement (*P* values were based on PERMANOVA). (D) Comparison of the unweighted UniFrac distance of 16S rRNA gene sequences between the two groups at different time points. For example, the first group of bars represents the comparison of unweighted UniFrac distances between SPF and Con groups at D0, D7, and D21. Error bars indicate standard error of mean (*n* = 9 or 10; *P* values were based on ANOVA with Sidak’s *post-hoc* test (variance is homogeneous) or ANOVA with Tamhane *post-hoc* test (variance is uneven); *P < 0.05, **P < 0.01, and ***P < 0.001).

We analyzed the unweighted UniFrac distance analysis of 16S rRNA gene sequences and calculated the differences among the groups by PERMANOVA. The results showed that the differences among the three groups after environmental hygiene improvement were greater than those before movement (Fig. 2A through C). The distance between the SPF and Con mice increased on the 7th and 21st day, and the difference was statistically significant (*P* = 3 × 10^−5^) compared with the day of environmental hygiene improvement (Fig. 2D). The unweighted UniFrac distance between the SPFL and Con groups increased significantly on the 7th and 21st day after environmental hygiene improvement (Fig. 2D). The unweighted UniFrac distance between the SPFL and SPF groups also significantly increased on the 7th and 21st day (Fig. 2D). In other words, the pairwise distances between any two groups were closest before the environmental change, and the improvement in environmental cleanliness increased the pairwise distances between the three groups. Analysis of the changes in the gut microbiota before and after environmental hygiene improvement revealed that the gut microbiota in the Con group were dynamic with time (Fig. S2A); however, the SPF and SPFL groups showed more significant changes after environmental hygiene improvement (Fig. S2).

To analyze genetic changes in the gut microbiota of mice after environmental hygiene improvement, 16S rRNA gene sequences were analyzed at the phylum levels. It was observed that the gut microbiota of SPF and SPFL mice exhibited greater alteration after environmental hygiene improvement than the gut microbiota of Con mice (Fig. S3). Specifically, environmental hygiene improvement increased the relative abundance of Deferribacteres and Actinobacteria in certain mice (Fig. S3).

### Environmental hygiene improvement causes the loss of a considerable number of gut microbes

Kruskal–Wallis test with Mann–Whitney U *post-hoc* test and random forest analysis was used to analyze the changes in gut microbiota in OTUs on the 21st day after environmental hygiene improvement. The results showed that 1,224 OTUs were significantly different between the SPF and Con groups (*P* < 0.05). A total of 1,002 OTUs were overrepresented in the Con group, and 222 OTUs were overrepresented in the SPF group (Fig. S4A; Table S4). Random forest results demonstrated that 434 OTUs could distinguish the gut microbiota between the SPF and Con groups (important score ≥0.0001), and 340 OTUs were overrepresented in the Con group (Fig. S4B; Table S4). Comparison between the SPFL and Con groups was similar to that of the SPF and Con groups (Fig. S4A and B; Table S3; Table S5). There were significant differences between the SPFL and SPF groups in 684 OTUs, of which 549 were overrepresented in SPF and 135 were overrepresented in the SPFL group (Fig. S4A; Table S4). Random forest analysis results showed that 212 OTUs could distinguish the gut microbiota of the SPFL and SPF groups, of which 140 were overrepresented in the SPF group (Fig. S4B; Table S5). We employed two distinct methods to analyze the differences in gut microbiota, thereby enhancing the reliability of our findings. Therefore, environmental hygiene improvement was associated with losing a substantial number of OTUs in the gut microbiota of mice (Fig. S5).

**Fig 3:**
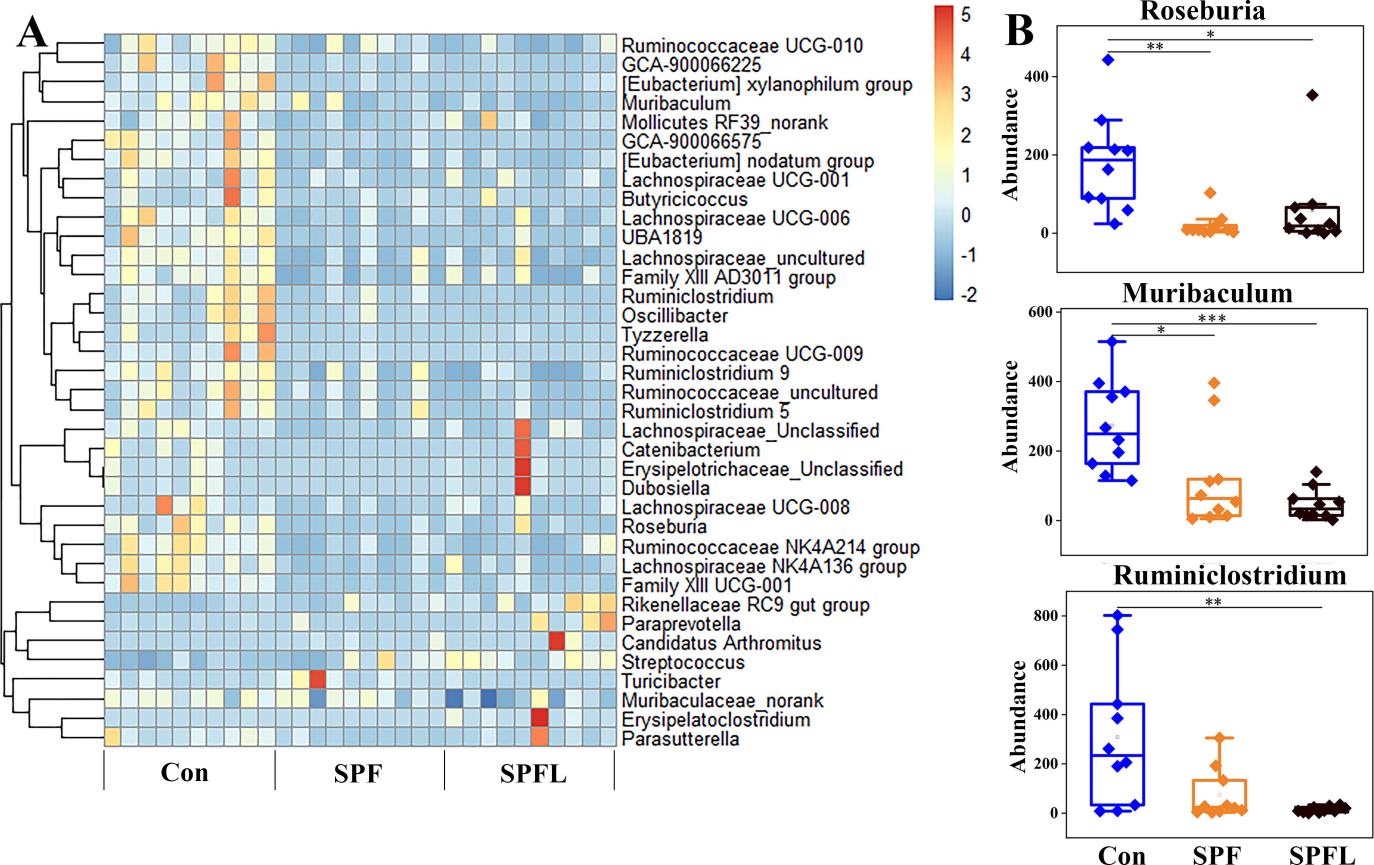
Differential analysis of gut microbiota at the genus level. (A) The heatmap displays the differential genera with a total abundance greater than 200 in all three groups (*P* < 0.05). All data sets are standardized with Z score. (B) Examples of differential genera from panel A. Error bars indicate standard error of mean. *P* values were calculated using the Kruskal–Wallis test with Mann–Whitney U *post-hoc* test (Bonferroni correction), with *P < 0.05, **P < 0.01, and ***P < 0.001.

**Fig 4:**
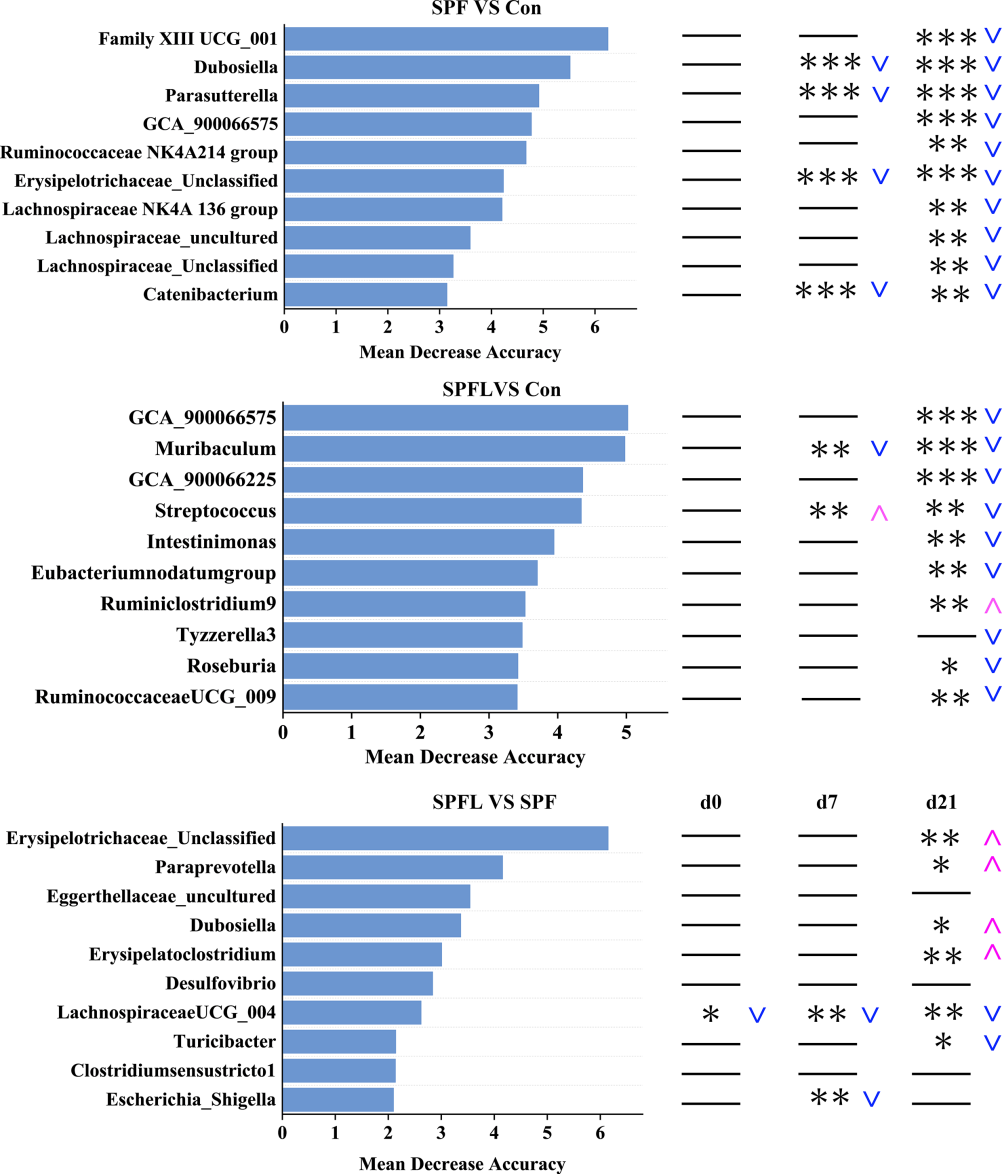
Random forest analysis of gut microbiota at the genus level. The top 10 genera are shown, ranked by the mean decrease accuracy index from random forest analysis at day 21 between the two groups. The subsequent panels display the differences in the abundances of these 10 genera between the two groups at D0, D7, and D21. *P* values were calculated using the Kruskal–Wallis test with Mann–Whitney U *post-hoc* test (Bonferroni correction), with *P < 0.05, **P < 0.01, and ***P < 0.001. Blue downward arrows indicate a decrease in the abundance in the previous group relative to the subsequent group, whereas magenta upward arrows indicate an increase in the abundance.

Due to the fact that many OTUs could not be annotated at the genus level during the OTU annotation process (see Table S4), we conducted a macro-level analysis of the overall changes in gut microbiota structure by directly analyzing OTU variations mentioned in the previous section. To further explore the specific taxonomic groups of the lost microorganisms, we performed a differential analysis at the genus level. The results showed that, with the increase in environmental hygiene, the abundance of most differential microorganisms decreased compared to that of the control group (Fig. 3A). These microorganisms include genera with protective effects against allergies, such as *Roseburia*, *Muribaculum*, and *Ruminiclostridium*, as well as short-chain fatty acid-producing genera such as *Lachnospiraceae NK4A136 group*, *Lachnospiraceae UCG-006*, and *Butyricicoccus* (Fig. 3A and B). Figure 4 shows the top ten genera ranked by the mean decrease accuracy index from random forest analysis. By analyzing the SPF and Con groups, we found that, before the improvement in environmental hygiene (D0), there was no significant difference between the two groups. At D7, the abundance of four genera in the SPF group was significantly lower than that in the Con group. By D21, the abundance of all ten genera in the SPF group was significantly reduced. The results from the SPFL group analysis were similar (Fig. 4).

**Fig 5:**
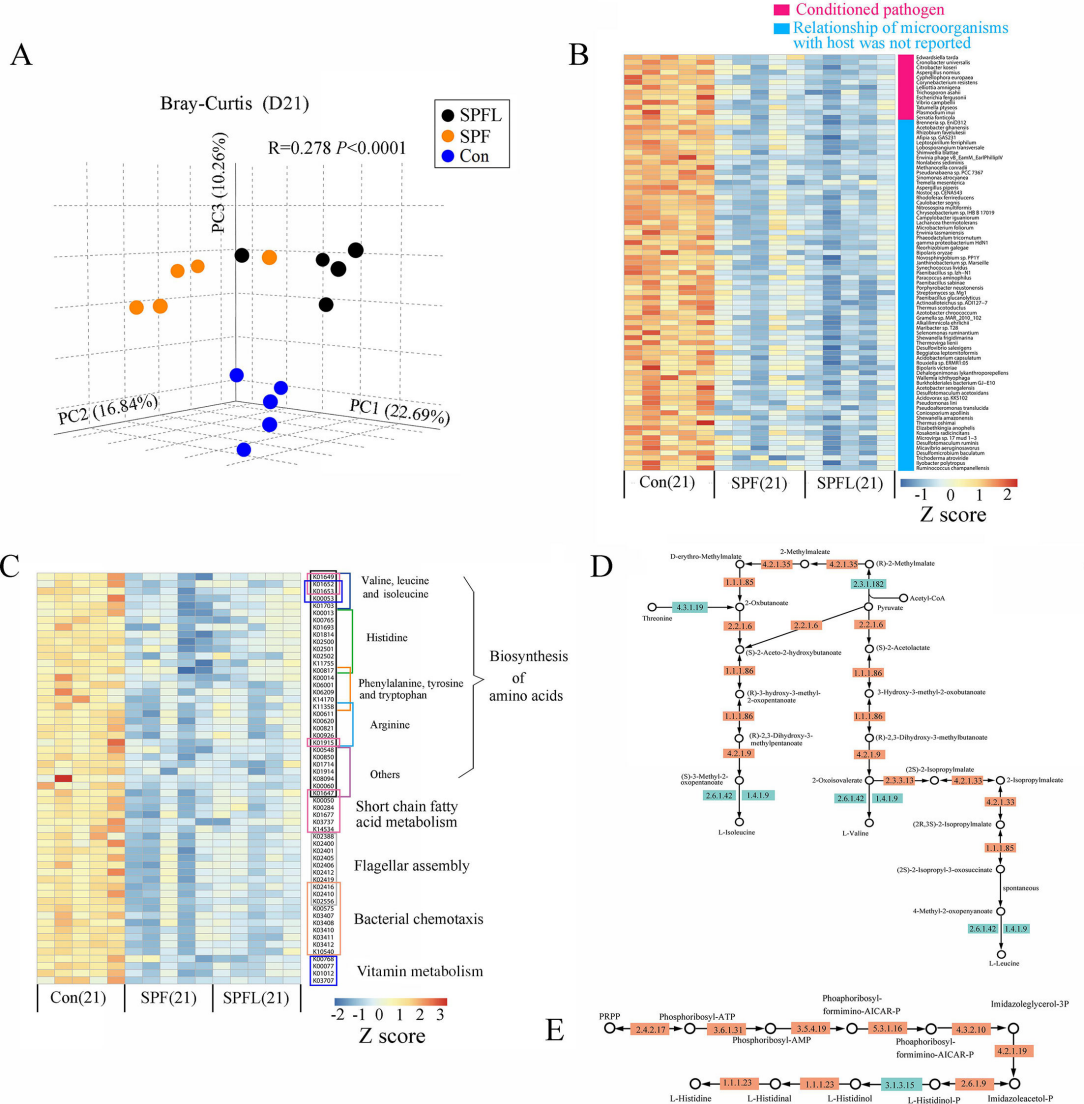
Analysis of shotgun sequencing of samples of the gut microbiome in the three experimental groups of mice on day 21 after environmental hygiene improvement. (A) Principal coordinate analysis (PCoA) of Bray–Curtis distance of shotgun sequencing on day 21 after environmental hygiene improvement (*P* values were based on PERMANOVA; ***P < 0.001). (B) Effects of environmental hygiene improvement on the gut microbiota in mice at the species level. All of them are presented in Table S6. (C) Effects of environmental hygiene improvement on functional genes of the gut microbiome in mice. The colors indicate the relative abundance. All data sets are standardized with Z score. Each column represents one mouse. (D) KEGG pathway for valine, leucine, and isoleucine biosynthesis. (E) KEGG pathway for histidine biosynthesis. In panels D and E, the orange color indicates functional genes are significantly higher in the Con compared to the SPF and SPFL groups. The light blue color represents functional genes that show no significant difference between the groups or were not detected during sequencing.

To analyze the effect of environmental hygiene improvement on the gut microbiome structure and expression of functional genes, we randomly selected five mice from each of the three groups on the 21st day after environmental hygiene improvement and performed shotgun sequencing analysis of the gut microbiome samples. The distance between two samples (dissimilarity) was calculated using the Bray–Curtis statistical method. Principal coordinate analysis (PCoA) results showed that the movement to a high-cleanliness environment led to changes in the gut microbiome of the SPF and SPFL mice compared with those of the Con group (Fig. 5A). Annotation of the shotgun sequencing data set showed data on 6,610 matched species. There were 1,186 significantly different species (*P* < 0.05) between the Con and SPF groups, of which 1,103 species were overrepresented in the Con group and 83 species were overrepresented in the SPF group (Fig. S4C; Table S6). The results of random forest analysis showed that 150 species could distinguish the gut microbiota between SPF and Con groups (important score ≥0.0001), of which 140 species were overrepresented in the Con group (Fig. S4D; Table S7). Comparison results between the SPFL and Con groups were similar to those between the SPF and Con groups (Fig. S4C and D; Tables S5 and S7). There were 97 species that showed significant differences between the SPF and SPFL groups, 62 of which were overrepresented in the SPF group (Fig. S4C; Table S6). Random forest analysis showed that 47 species could be distinguished in the gut microbiota between SPFL and SPF groups, and 28 species were overrepresented in the SPF group (Fig. S4D; Table S7). Based on the above-mentioned analysis, we concluded that environmental hygiene improvement depleted many species in the mouse gut microbiota. This observation followed the results of 16S rRNA gene sequencing. We conducted a search of the PubMed database to investigate the effects of these differential microorganisms on the host. We found that the majority of these microorganisms have not been reported in relation to the host. Among the remaining microorganisms that have been reported, most are opportunistic pathogens (Fig. 5B). The reason for this may be that current research primarily reports the functions of gut microbiota at the genus level.

In summary, the increase in environmental cleanliness has resulted in a significant loss of a large number of gut microbiota, including bacteria that play a protective role against allergies and those that produce short-chain fatty acids.

### Environmental hygiene improvement causes the loss of a large number of functional genes in the gut microbiome

To explore the effects of environmental hygiene improvement on functional genes of the gut microbiome in the mice, we annotated the function of the shotgun sequences using the Kyoto Encyclopedia of Genes and Genomes (KEGG) database. We compared the differences between the SPF and Con groups and between the SPFL and Con groups by Kruskal–Wallis test with Mann–Whitney U *post-hoc* test. The results identified that 32 enzyme genes related to the synthesis of amino acids, including valine, leucine, isoleucine, tryptophan, and other essential amino acids, had significantly lower expression levels in the SPF and SPFL groups after environmental hygiene improvement with high cleanliness compared with those in the Con group (Fig. 5C through E; Fig. S6; Table S8). Additionally, we found seven genes related to vitamin metabolism, 10 genes related to short-chain fatty acid metabolism, 10 genes related to bacterial chemotaxis, and 10 genes related to flagellar assembly, which showed significantly lower expression levels in the SPF and SPFL groups compared to those in the Con group (Fig. 5C; Table S8). This indicated that a large number of functional genes in the gut microbiome of the mice were lost after environmental hygiene improvement.

### Environmental hygiene improvement aggravates chronic inflammatory dermatosis in DNFB-treated mice

Several studies have reported the importance of gut microbiota on the development of the immune system in early stages of life (34–36), but few studies have reported the impact of gut microbial changes caused by environmental hygiene improvement on allergic diseases in adulthood. Repeated local use of DNFB can induce the clinical symptoms of chronic inflammatory dermatosis in mice (19). To investigate whether changes in gut microbiota of mice caused by environmental hygiene improvement in adulthood affected immune homeostasis, we used DNFB to treat the skin and ears of the mice at 21 weeks of age, 13 weeks after movement.

**Fig 6:**
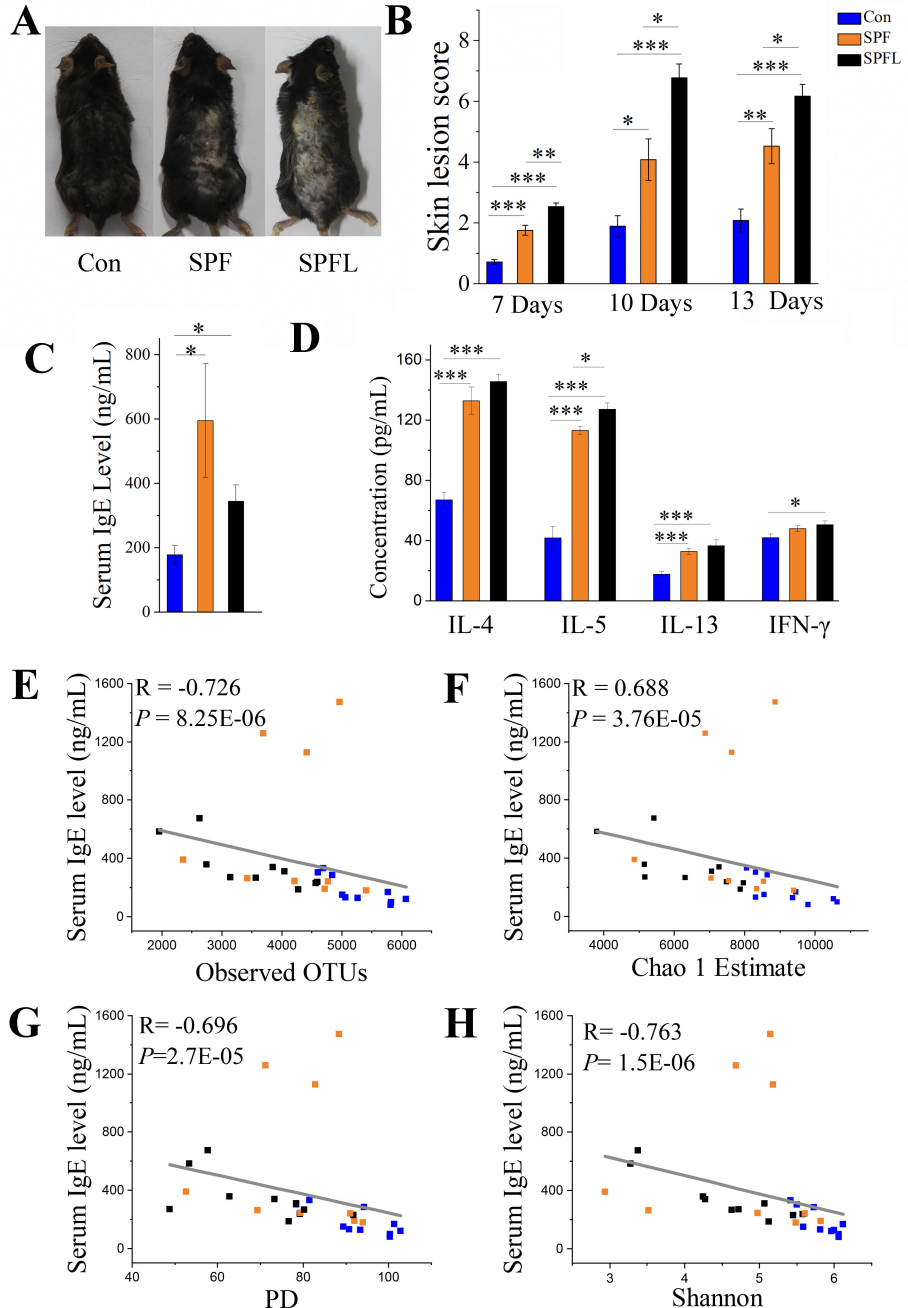
Effects of environmental hygiene improvement on the immune function of mice. (A) Representative images of mouse skin taken on the 14th day after sensitization. (B) Skin lesion scores on days 7, 10, and 13 after sensitization (*n* = 9 or 10; *P* values were based on ANOVA with Sidak’s *post-hoc* test (variance is homogeneous) or ANOVA with Tamhane *post-hoc* test (variance is uneven). (C) Serum IgE levels on the 14th day after sensitization (*n* = 9 or 10; *P-*values are based on Kruskal–Wallis test with Mann–Whitney U *post-hoc* test). (D) Levels of IL-4, IL-5, and IL-13 in serum (*n* = 8; *P* values were based on ANOVA with Sidak’s *post-hoc* test (variance is homogeneous) or ANOVA with Tamhane *post-hoc* test (variance is uneven)). (E–H) Spearman’s correlation between serum IgE level and gut microbial diversity. Error bars indicate standard error of mean. *P < 0.05, **P < 0.01, and ***P < 0.001.

Skin lesions were scored, and serum IgE and cytokine levels were evaluated. Skin lesions of the SPF and SPFL groups were more remarkable than those of the control group, and the lesions exhibited a gradient change with increased environmental cleanliness (Fig. 6A and B). Serum IgE, IL-4, IL-5, and IL-13 levels of the SPF and SPFL mice were significantly higher compared to those in the Con mice (Fig. 6C and D). In addition, IFN-γ, IL-2, and IL-6 showed an increasing trend in the SPF and SPFL mice, with IFN-γ in the SPFL being significantly elevated compared to the Con group (Fig. 6D and S7). Therefore, environmental hygiene improvement increased the skin inflammation of adult mice treated with DNFB.

Associations between gut microbial diversity (Shannon index, Chao1 estimates, observed OTUs, and PD index) and serum IgE levels were investigated by calculating Spearman’s correlation coefficients. It was found that there was a significant negative correlation between serum IgE levels and gut microbial diversity (R < −0.6, *P* < 0.0001, Fig. 6E through H).

## DISCUSSION

In this study, mice were used to explore the effects of environmental hygiene improvement on the gut microbiome. The results showed that environmental hygiene improvement decreased gut microbial diversity and reduced a large number of microbes and functional genes. The lost microbiota includes bacteria that play a protective role against allergies and those that produce short-chain fatty acids. Furthermore, total serum IgE and cytokine (IL-4, IL-5, and IL-13) levels of mice with increased environmental cleanliness were significantly higher compared to those of control mice following induction of skin allergy using DNFB. Comparing SPF and SPFL, preventing mice from eating feces decreased the gut microbial diversity, but no significant change was observed in the abundance of functional genes.

Environmental hygiene improvement significantly decreased the diversity of the gut microbiota in mice and shaped the microbial composition. Vangay et al. found that dietary changes can partially explain the decrease in gut microbial diversity after relocating to developed countries from developing countries (2). From this study, it can be seen that environmental hygiene improvement may also play an important role in the changes in gut microbiota.

Many studies have indicated that the cleanliness of the living environment in early stages of life influences the colonization of gut microbiota (37) and an unclean living environment increases the diversity of gut microbes in children and shapes the microbial composition (10, 38). The impact of low-cleanliness environments on the gut microbiota of adults, particularly changes in cleanliness, has received limited attention and research. However, this study showed that alterations in the cleanliness of living environments in adult mice have a significant impact on the structural composition of gut microbiota.

The current results showed that environmental hygiene improvement aggravated chronic inflammatory dermatosis in mice. This may partly explain the reason for increased incidences of allergic diseases in people moving from developing countries to developed countries (9). There was a significant negative correlation between serum IgE levels and gut microbial diversity in migrated mice. The “old friends” hypothesis proposes that human microorganisms have coevolved with humans, and some of them have played a beneficial role in the development of our immune systems. However, with urbanization, many of these microorganisms have become less prevalent or even completely absent (39).

Relocation from developing countries to developed countries increases the incidence rate of allergies and other chronic diseases (2, 9). This trend may be related to the loss of large gut microbiota, which subsequently affects immune tolerance levels. In this study, the abundance of *Roseburia*, *Muribaculum*, and *Ruminiclostridium* significantly decreased in both groups of mice exposed to increased environmental cleanliness. Several studies have indicated that *Roseburia* has a protective role against allergies (40–42), with Tamanai-Shacoori et al. even reporting that *Roseburia* can serve as a marker of health (41). *Muribaculum* and *Ruminiclostridium* have also been reported to reduce the risk of allergies (43, 44). The reduction in the abundance of these allergy-protective bacteria due to the increase in environmental cleanliness may partly explain the exacerbation of chronic inflammatory dermatosis observed in this study.

With the increase in environmental cleanliness, *Lachnospiraceae NK4A136 group*, *Lachnospiraceae UCG-006*, and *Butyricicoccus* were lost. These bacteria have been reported to have beneficial effects on health by producing short-chain fatty acids (45–48). Shotgun sequencing analysis showed that the abundance of functional genes related to amino acid synthesis, short-chain fatty acid metabolism, and vitamin B1 and B12 metabolism significantly decreased after environmental hygiene improvement. It is well known that the above-mentioned metabolites play a role in maintaining immune homeostasis, especially short-chain fatty acids (49, 50). Trompette et al. found that the circulating levels of short-chain fatty acids in the gut were associated with allergic inflammation in the lungs (51). Furthermore, these metabolites also function in maintaining the homeostasis of the gut microecology and for providing nutrition to the host (49, 50).

In this study, preventing mice from eating feces reduced the gut microbial diversity and altered the structure of the gut microbiota. It is suggested that eating feces is an important factor to maintain the stability of gut microbiota in mice. The possible reason is that eating feces can also result in intake of microbes and prebiotics in feces. Mice are important animal models for studying human diseases, and human beings are strictly separated from feces. The experimental results of mice preventing fecal ingestion may be closer to those of human beings.

The results of this study are consistent with those of most studies, but a few studies have reported contrasting results. For example, Ma et al*.*’s findings suggest that mice with wild-type gut microbiota exhibit more severe allergic asthma compared to SPF mice (52). The discrepancy may arise from the fact that gut microbiota is an extremely complex and dynamically balanced ecosystem. Wild-type mice raised using different methods in different laboratories have distinct gut microbiota. These variations may lead to contradictory results across different experiments. This underscores the need for more advanced methods and analytical techniques to better understand what constitutes a healthy gut microbiota.

The results of this study indicate that increased environmental cleanliness reduces the diversity of gut microbiota in adult mice and exacerbates the severity of DNFB-induced chronic inflammatory dermatosis. This study established a mouse model to investigate how changes in environmental hygiene impact gut microbiota and, consequently, immune responses. Based on this model, future studies should integrate metabolomics approaches to identify lost metabolites at the protein level. Additionally, supplementation with these metabolites or relevant microbes in germ-free mice could be used to explore their precise effects on immune responses, thereby providing insights for translational research.

Overall, changes in the human living environment are accompanied by alterations in various factors that can impact the gut microbiota, such as the use of antibiotics and other medications, changes in diet, stress, and exercise. Therefore, animal experiments are needed to validate the impact of changes in a single factor on the gut microbiota and related diseases. However, animal experiments are distinct from human studies, and the number of animals used in this study was limited. Therefore, the interpretation of the impact of increased environmental cleanliness on human gut microbiota and inflammatory symptoms based on these experimental results is speculative. More research is needed in the future to validate the underlying mechanisms.

## Data Availability

Metagenomic sequence data for each mouse are available in the NCBI Sequence Read Archive under accession number PRJNA686215.

## SUPPLEMENTAL MATERIAL

Supplemental figures aem.02477-24-s0001.pdf Figures S1 to S7.

Table S1 aem.02477-24-s0002.xlsx Description of soil added to bedding of mice in the general animal room.

Table S2 aem.02477-24-s0003.xlsx Sample information.

Table S3 aem.02477-24-s0004.xlsx Comparison of α diversity between groups based on 16S rDNA sequencing.

Table S4 aem.02477-24-s0005.xlsx Comparison of operational taxonomic units (OTUs) between groups based on 16S rDNA sequencing.

Table S5 aem.02477-24-s0006.xlsx Operational taxonomic units (OTUs) identified by random forest analysis of 16S rDNA sequencing results that exhibit significant differences between groups.

Table S6 aem.02477-24-s0007.xlsx Comparison of species between groups based on shotgun sequencing.

Table S7 aem.02477-24-s0008.xlsx Species identified by random forest analysis of shotgun sequencing results that exhibit significant differences between groups.

Table S8 aem.02477-24-s0009.xlsx Environmental hygiene improvement leads to the loss of a large number of functional genes in the gut microbiome.

ASM does not own the copyrights to Supplemental Material that may be linked to, or accessed through, an article. The authors have granted ASM a non-exclusive, world-wide license to publish the Supplemental Material files. Please contact the corresponding author directly for reuse.
