# Supplementary material for: Effects of post-adulthood environmental hygiene improvement on gut microbiota and immune tolerance in mice
Source: Appl Environ Microbiol. 2025 Mar 6;91(4):e02477-24. doi: 10.1128/aem.02477-24 (PMC12016539; doi:10.1128/aem.02477-24)
Supplement: Supplemental figures — Figures S1 to S7. [file aem.02477-24-s0001.pdf]

Supplementary Information

Effects of Post-Adulthood Environmental Hygiene Improvement on Gut Microbiota and Immune Tolerance in Mice

This file includes:

Figures S1 to S7

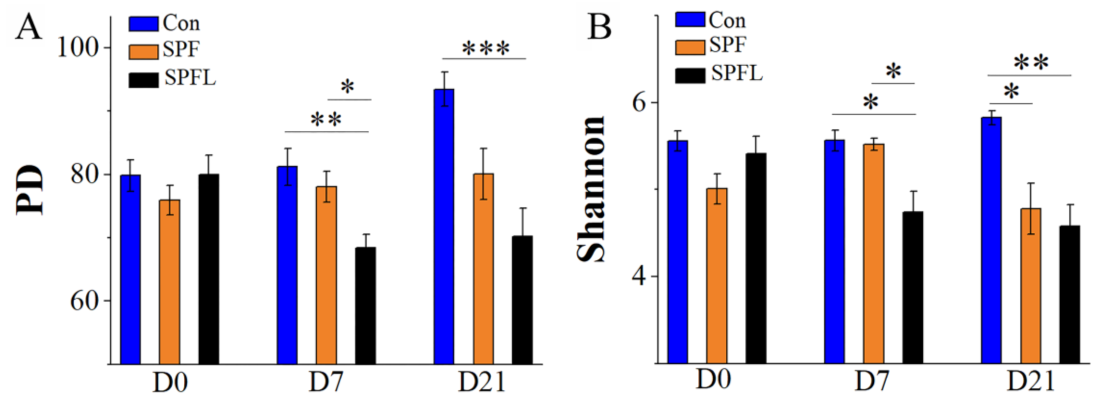

Figure S1. Effects of environmental hygiene improvement on the diversity of gut microbiota in mice. A. Comparison of phylogenetic diversity (PD) index among the different experimental groups at the same time point. B. Comparison of the Shannon diversity index among the different groups of experimental mice at the same time point. (n = 9 or 10; Error bars indicate standard error of mean. P-values were based on ANOVA with sidak post-hoc test (variance is homogeneous) or ANOVA with Tamhane post-hoc test (variance is uneven); *$P < 0.05$, **$P < 0.01$, ***$P < 0.001$)

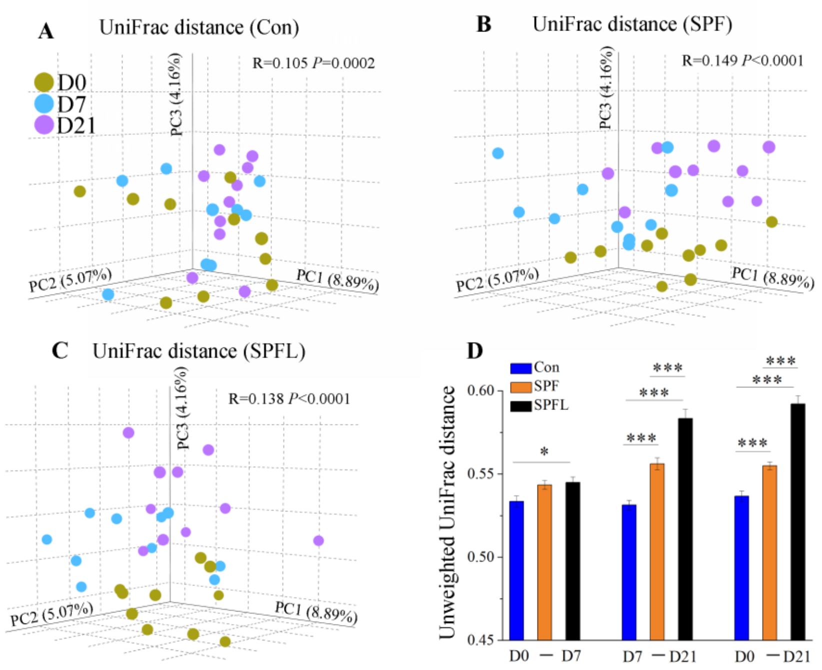

Figure S2. Principal coordinate analysis (PCoA) of unweighted UniFrac distance of the gut microbiota in mice before and after environmental hygiene improvement. A. Con group. B. SPF group. C. SPFL group. *P*-values were based on PERMANOVA. D. Comparison of unweighted UniFrac distance of 16S rRNA gene sequences over time between different groups. For example, the first group of bars represents the comparison of changes in unweighted UniFrac distance from D0 to D7 among the three groups. Error bars indicate standard error of mean, P values were based on ANOVA with sidak post-hoc test (variance is homogeneous) or ANOVA with Tamhane post-hoc test (variance is uneven). **P* < 0.05, ***P* < 0.01, ****P* < 0.001.

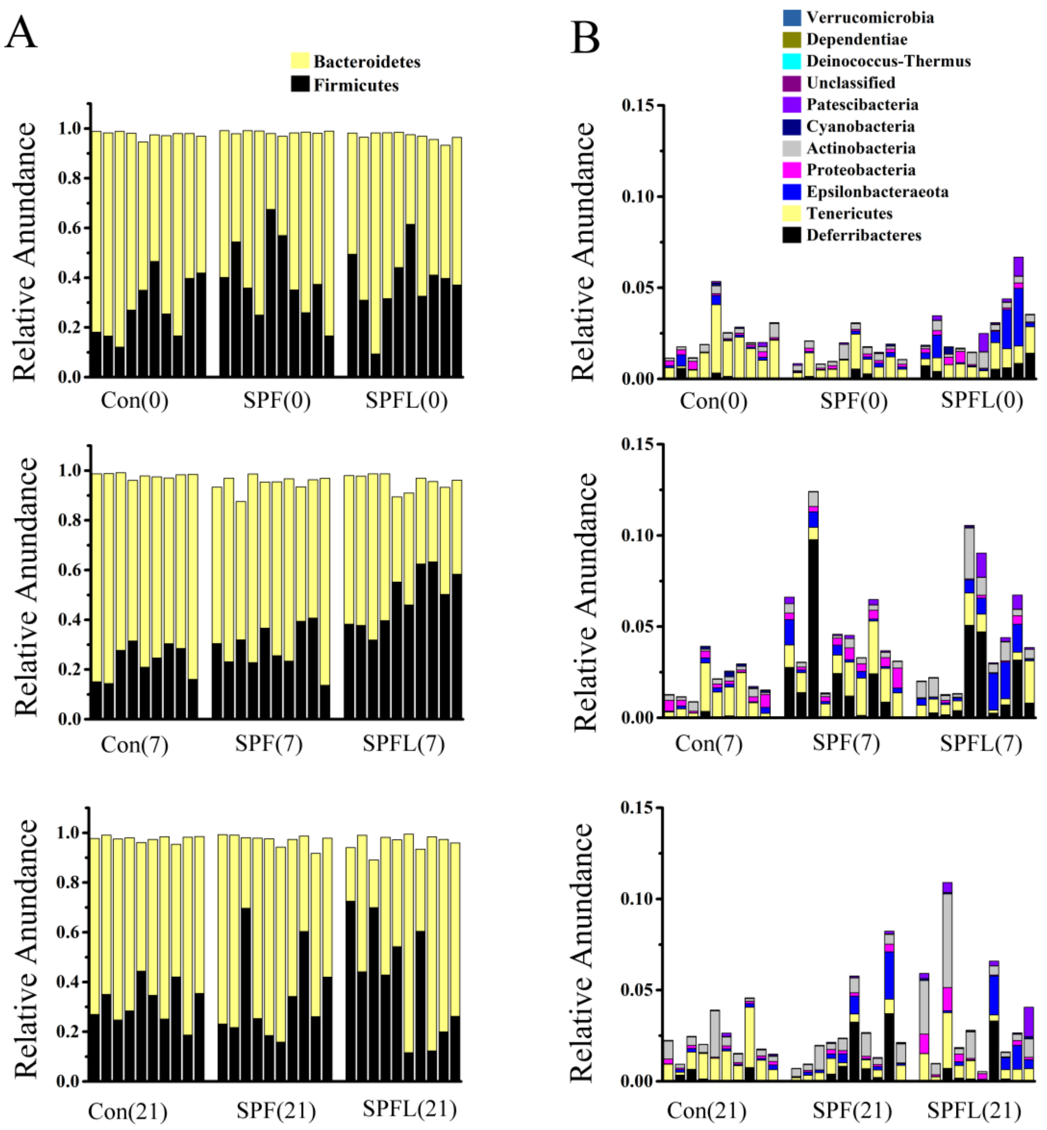

Figure S3. Effect of environmental hygiene improvement on the gut microbiota in mice at the phylum level.

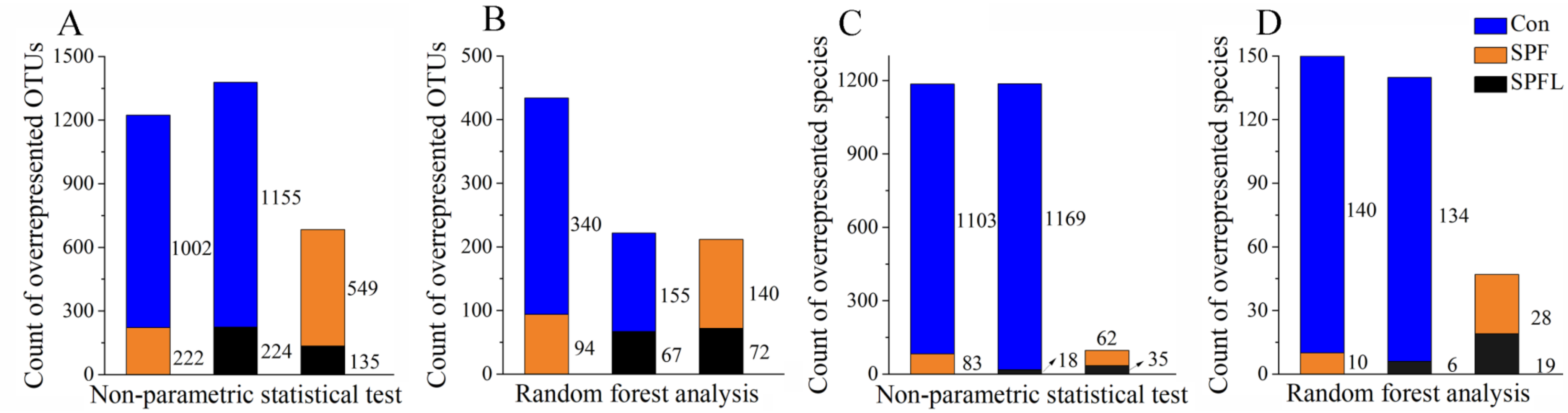

Figure S4. Effect of environmental hygiene improvement on the gut microbiota in mice at the OTU and species level. A Comparison of operational taxonomic units (OTUs) between groups based on 16S rRNA gene sequencing. Kruskal-Wails test with Mann-Whitney U post-hoc test (bonferroni correction) was used to analyze the difference. The figure shows the count of OTUs with P value less than 0.05. B. Operational taxonomic units (OTUs) identified by random forest analysis of 16S rRNA gne sequencing. Random forest analysis assigns an importance score to each OTU by estimating the increase in error caused by removing a specific OTU from the set of predictors. The figure shows the count of OTUs with importance score greater than 0.0001. C. Comparison of species between groups based on shotgun sequencing. Kruskal-Wails test with Mann-Whitney U post-hoc test (bonferroni correction) was used to analyze the difference. The figure shows the count of species with P value less than 0.05. D. Species identified by random forest analysis of shotgun sequencing. Random forest analysis assigns an importance score to each species by estimating the increase in error caused by removing a specific species from the set of predictors. The figure shows the count of species with importance score greater than 0.001.

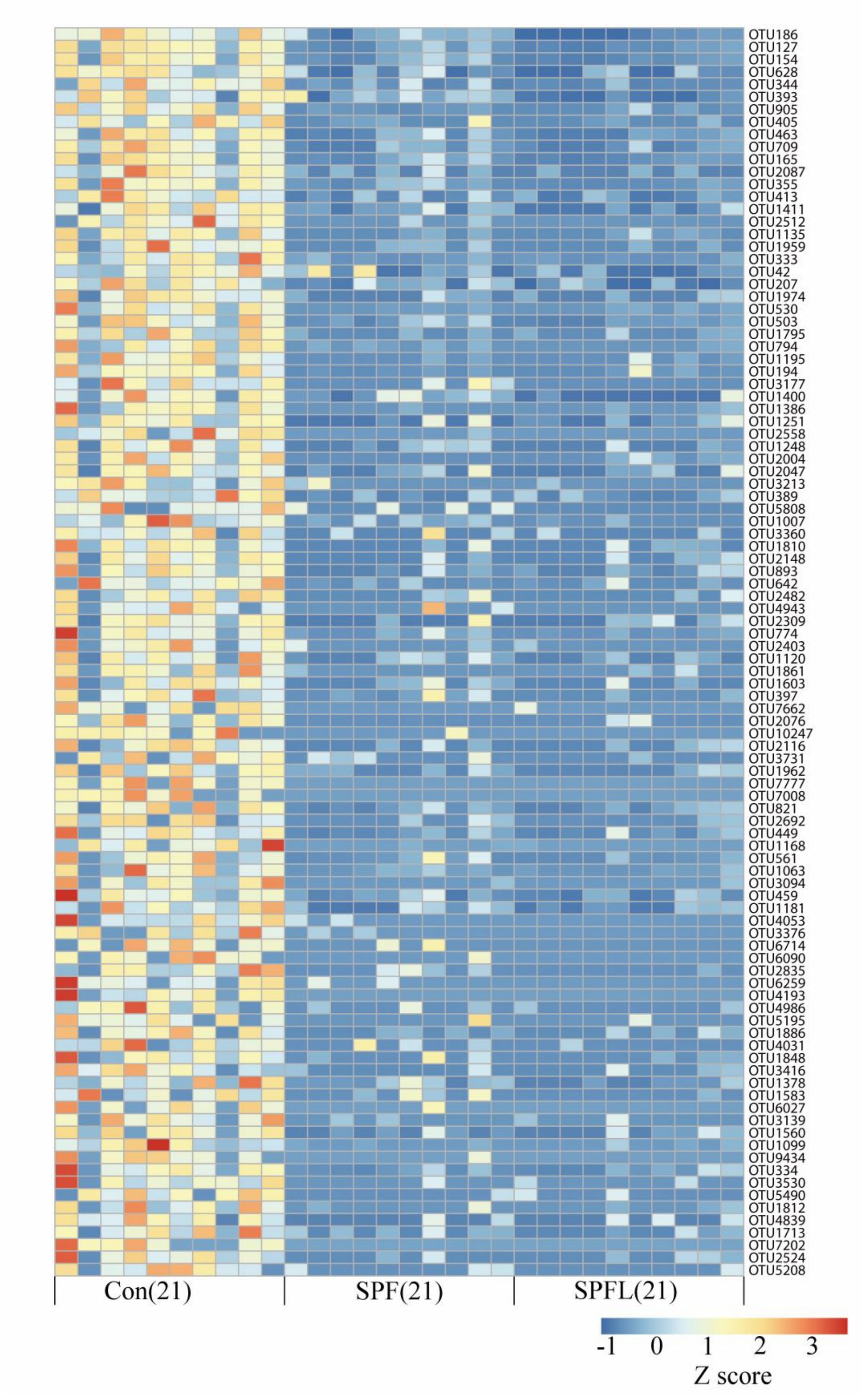

Figure S5. Effects of environmental hygiene improvement on operational taxonomic units (OTUs) of the gut microbiome in mice. All data sets are standardized with Z score.

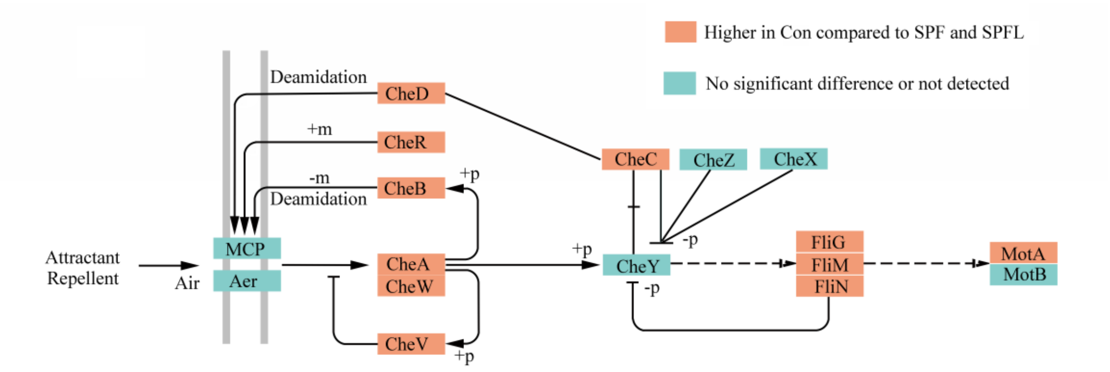

Figure S6. Kyoto Encyclopedia of Genes and Genomes (KEGG) pathway for bacterial chemotaxis.

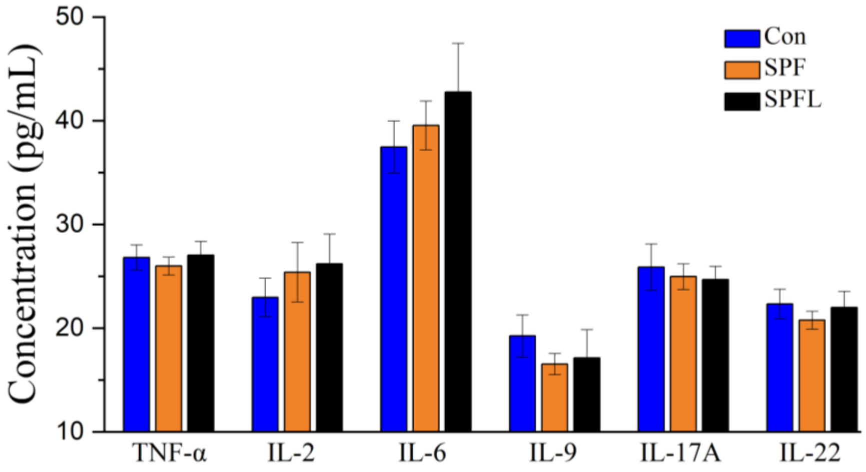

Figure S7. Levels of cytokine in serum (n = 8; P values were based on ANOVA with sidak post-hoc test (variance is homogeneous) or ANOVA with Tamhane post-hoc test (variance is uneven), Error bars indicate standard error of mean).
